# Development and Validation of a Machine Learning Model to Estimate Risk of Adverse Outcomes Within 30 Days of Opioid Dispensation

**DOI:** 10.1001/jamanetworkopen.2022.48559

**Published:** 2022-12-27

**Authors:** Vishal Sharma, Vinaykumar Kulkarni, Ed Jess, Fizza Gilani, Dean Eurich, Scot H. Simpson, Don Voaklander, Michael Semenchuk, Connor London, Salim Samanani

**Affiliations:** 1 Li Ka Shing Center for Health Research Innovation, School of Public Health, University of Alberta, Edmonton, Alberta, Canada; 2 OKAKI, Calgary, Alberta, Canada; 3 College of Physicians and Surgeons of Alberta, Edmonton, Alberta, Canada; 4 Faculty of Pharmacy and Pharmaceutical Sciences, University of Alberta, Edmonton, Alberta, Canada; 5 School of Public Health, University of Alberta, Edmonton, Alberta, Canada

## Abstract

**Importance:**

Machine learning approaches can assist opioid stewardship by identifying high-risk opioid prescribing for potential interventions.

**Objective:**

To develop a machine learning model for deployment that can estimate the risk of adverse outcomes within 30 days of an opioid dispensation as a potential component of prescription drug monitoring programs using access to real-world data.

**Design, Setting, and Participants:**

This prognostic study used population-level administrative health data to construct a machine learning model. This study took place in Alberta, Canada (from January 1, 2018, to December 31, 2019), and included all patients 18 years and older who received at least 1 opioid dispensation from a community pharmacy within the province.

**Exposures:**

Each opioid dispensation served as the unit of analysis.

**Main Outcomes and Measures:**

Opioid-related adverse outcomes were identified from administrative data sets. An XGBoost model was developed on 2018 data to estimate the risk of hospitalization, an emergency department visit, or mortality within 30 days of an opioid dispensation; validation on 2019 data was done to evaluate model performance. Model discrimination, calibration, and other relevant metrics are reported using daily and weekly predictions on both ranked predictions and predicted probability thresholds using all data from 2019.

**Results:**

A total of 853 324 participants represented 6 181 025 opioid dispensations, with 145 016 outcome events reported (2.3%); 46.4% of the participants were men and 53.6% were women, with a mean (SD) age of 49.1 (15.6) years for men and 51.0 (18.0) years for women. Of the outcome events, 77 326 (2.6% pretest probability) occurred within 30 days of a dispensation in the validation set (XGBoost *C* statistic, 0.82 [95% CI, 0.81-0.82]). The top 0.1 percentile of estimated risk had a positive likelihood ratio (LR) of 28.7, which translated to a posttest probability of 43.1%. In our simulations, the weekly measured predictions had higher positive LRs in both the highest-risk dispensations and percentiles of estimated risk compared with predictions measured daily. Net benefit analysis showed that using machine learning prediction may not add additional benefit over the entire range of probability thresholds.

**Conclusions and Relevance:**

These findings suggest that prescription drug monitoring programs can use machine learning classifiers to identify patients at risk of opioid-related adverse outcomes and intervene on high-risk ranked predictions. Better access to available administrative and clinical data could improve the prediction performance of machine learning classifiers and thus expand opioid stewardship efforts.

## Key Points

**Question:**

Can a machine learning model trained on available administrative data estimate adverse opioid outcomes for prescription drug monitoring programs?

**Findings:**

In this prognostic study of 853 324 adults in Alberta, Canada, administrative data (145 016 reported outcome events for 2018-2019) were used to construct a machine learning model to estimate adverse outcome risk within 30 days of opioid dispensation. High model prediction performance was observed, with 3% pretest probability compared with 43% posttest probability.

**Meaning:**

These findings suggest that with comprehensive access to routinely collected administrative health data, machine learning can help prescription drug monitoring programs identify and intervene in cases of high-risk opioid prescribing to fulfill opioid stewardship mandates.

## Teaser

This prognostic study uses machine learning to estimate the risk of adverse outcomes within 30 days of opioid dispensation in Alberta, Canada, as a potential component of prescription drug monitoring programs to identify and intervene in cases of high-risk opioid prescribing.

## Introduction

Canada experiences some of the highest rates of opioid prescribing in the world, making prescription opioid use a key driver of the opioid crisis.^1^ The consequences of prescribed opioids are well characterized.^1,2,3,4^ As part of the response to the opioid crisis, health jurisdictions and regulators have implemented prescription drug monitoring programs (PDMPs) such as the Tracked Prescription Program (TPP) Alberta administered by the College of Physicians and Surgeons of Alberta (CPSA).^5^ The CPSA, like many other health regulators, has a mandate to protect Albertans by guiding the medical profession and plays a major role in prescription opioid stewardship.

With the increase in digital health, there is a growing movement to integrate emerging digital technologies (eg, machine learning [ML]) with medicine.^6,7,8^ Health regulators like the CPSA are leading this trend by using data to optimize patient safety.^5^ The Government of Alberta maintains a comprehensive infrastructure of administrative health data, and the CPSA has limited access to certain data sets—namely, community pharmacy prescription dispensation records.

Supervised ML^6^ is an approach that uses computer algorithms and the large amounts of available administrative data to build clinical prediction models, all within a well-defined framework.^9^ Supervised ML trains on labeled data to develop prediction models that are specific to different populations, and there are numerous published studies describing its use in clinical settings.^10,11,12^ However, reporting of ML prediction performance metrics is still inconsistent in the literature,^12^ although guidelines are in the works.^13^ To date, we are not aware of any health jurisdictions in Canada that use ML approaches in their opioid stewardship programs.

Others have described ML methods to estimate adverse outcomes pursuant to an opioid dispensation. For example, Lo-Ciganic et al^10^ assessed risk in Medicare beneficiaries and constructed ML models at the opioid dispensation level. Their findings support ML methods using administrative data to be a valuable tool for assessing risk in patients taking opioids. Lo-Ciganic et al^10^ also had access to more extensive data sets compared with the CPSA and presented high-performing ML prediction findings in a more general setting than what we propose.

Building on our previous work,^14^ the objective of this study was to develop and validate a proof-of-concept XGBoost^15,16^ ML model for use by the CPSA that could estimate the risk of adverse outcomes within 30 days of an opioid dispensation in Alberta, Canada. The ML model was trained only on prescription drug records to simulate a potential ML classifier deployment by the CPSA that is aligned with the type of data to which TPP Alberta has access. We evaluated the ML model using the same performance metrics^13,17,18,19^ as in our previous work and provide a general description of a customizable online dashboard. Using the CPSA as an example, our analysis provides interested prescription regulators with analytic options to assess the value and implementation of the ML classifier based on workload capacity. Although our study was conducted for the CPSA using data from Alberta, the ML process allows for others in different jurisdictions to deploy their own population- and data-specific ML risk classifiers. We propose that our ML classifier trained on local population-level data can provide predictions that are helpful to opioid stewardship mandates.

## Methods

This prognostic study was approved by the University of Alberta Ethics Board. Participant consent was waived owing to the use of deidentified data. The study followed the Transparent Reporting of a Multivariable Prediction Model for Individual Prognosis or Diagnosis (TRIPOD) guideline and other published guidelines^6,12^ specific to ML projects.

### Study Design, Setting, and Participants

This study used a supervised ML approach that trained an XGBoost model on opioid dispensations between January 1, 2018, and December 31, 2019, in Alberta, Canada. We used XGBoost due to it producing the highest prediction performance based on our previous work^14^ and also because it generates an interpretable model. We included all Albertans who were 18 years or older and had received at least 1 opioid dispensation from a community pharmacy. Because the CPSA only has access to certain drug history data (eg, medication dispensations) and not comorbidity, diagnoses, or laboratory data, we could not exclude any patients based on comorbidities.

### Data Sources

Although a wide range of administrative databases are available in Alberta, few are readily available to professional regulatory agencies like the CPSA, a common issue in many jurisdictions. Indeed, the CPSA currently only has access to prescription drug records in their role as administrator of TPP Alberta. Thus, we limited the data sets used to train the ML model to those accessible by the CPSA. Health regulators in other jurisdictions may have access to additional data to train ML models and create their own specific ML classifier.

To train the ML model, we used data from the Pharmaceutical Information Network (PIN), which has comprehensive information on dispensing records from community pharmacies in Alberta irrespective of coverage status and age.^20,21^ The PIN data were further filtered using Anatomical Therapeutic Chemical (ATC) classification system codes to include only those prescription records for which the CPSA has access.^5^ The CPSA receives records of opioid, benzodiazepine, and antibiotic dispensations in daily updates from Alberta Health.

To label each instance of an opioid dispensation with an outcome, we linked the prescription data with (1) population and vital statistics data and (2) hospitalizations and emergency department (ED) visits. Population and vital statistics data were obtained from the Service Alberta Vital Statistics Office (VS) and included the following: sex, age, date of birth, immigration and emigration data within the province, death date, and underlying cause of death according to the World Health Organization algorithm using *International Statistical Classification of Diseases and Related Health Problems, 10th Revision* (*ICD-10*) codes.^22^ Data on hospitalizations and ED visits were obtained from the National Ambulatory Care Reporting System (NACRS) and the Discharge Abstract Database (DAD) and included the following: all services, length of stay, and diagnoses (up to 25 *ICD-10*–based diagnoses). Data and coding accuracy are routinely validated both provincially and centrally via the Canadian Institute for Health Information.^23^

These linked databases represent a population-level labeled data set used to develop the XGBoost model. Currently, the CPSA does not have access to outcomes data (eg, DAD, NACRS, and VS) or physician claims data. We labeled data equivalent to the PIN data that the CPSA would receive with DAD, NACRS, and VS data to assess a proof-of-concept ML model.

### Measures and Outcomes

In this study, the unit of analysis was at the opioid dispensation level, such that each opioid dispensation was treated as independent and served as a potential instance to estimate our outcome. We chose this level of analysis to be consistent with others,^10^ to have more data to train the ML model, and to accurately represent real-use case scenarios in which a health regulator’s PDMP may want to assess the risk for each instance rather than a single or random dispensation. We identified opioid dispensations from the PIN file using ATC codes^24^ (eTable 1 in Supplement 1).

Our outcome was a composite of a drug-related ED visit, hospitalization, or death within 30 days of an opioid dispensation based on *ICD-10* codes from the linked databases (eTable 2 in Supplement 1).^25^ One-month risk windows are commonly used by health systems for risk assessments.^26^ Follow-up and predictions started after each opioid dispensation.

Rare outcomes are common in clinical prediction model development, and we anticipated this study to be no different than others^10^ because our training data would be class imbalanced. To address this issue, we applied the frequently used class weightage method, which does not alter the data distribution and instead increases the importance of the positive class (instances that led to the outcome).^27^

### Predictors and ML Methods

All candidate predictors were obtained from the PIN data set and were either derived from the literature^2,28^ or directly incorporated from the data unmodified (eTable 1 in Supplement 1). These features included demographic information (age, sex, Forward Sortation Index from postal codes,^29^ and income), medication use (ATC codes, oral morphine equivalents,^30^ concurrent use with benzodiazepines, number of dispensations, and number of unique molecules), and health care use (number of opioid prescribers and pharmacies); the eAppendix in Supplement 1 includes a complete list of features. We used data 30 days before each index opioid dispensation to measure each predictor; all features were measured up to and including the index opioid dispensation date from which the 30-day prediction follow-up period started (eFigure 2 in Supplement 1). Our research partners at the CPSA specified use of 30-day time periods. However, other health regulators can specify whichever time period they feel is important for their PDMPs; we did evaluate the potential impact of a longer window of 90 or 180 days, but it did not change our model performances and is otherwise not presented.

We used XGBoost to train our ML model. The opioid dispensations in 2018 were included in the development set, while those in 2019 were used for validation. We performed cross-fold validation (k = 5) in the development set to tune hyperparameters. With XGBoost, we tuned for tree height, number of trees, and weight scaling to address class imbalance.^15,16^

The validation set was defined from all of the 2019 dispensations and thus represents temporal validation. Participants in the development set could also be in the validation set, as this represents the real-world scenario in which health regulators work where patient instances are repeatedly encountered; indeed, it is ideal to develop an ML prediction model trained on data from the population in which it will be deployed (eFigure 1 in Supplement 1).

### Statistical Analysis

Using χ^2^ and *t* tests, we first described characteristics and outcome event rates in the development vs validation groups and between those who experienced the outcome and those who did not. This descriptive analysis was done at the patient level, in which each patient could be represented by multiple instances of opioid dispensations. We included information on age, sex, number of participants, and health care use.

The validation set was used to evaluate our ML model’s prediction performance with metrics that are commonly applied to clinical prediction models.^13,17,18,19^ As is done in many ML prediction studies,^12^ we assessed our XGBoost model’s discrimination performance by calculating the area under the receiver operating characteristic (AUROC) curve. For binary classification studies like ours, AUROC curves correspond to *C* statistics, which are a measure of model discrimination performance—the extent to which a model predicts a higher probability of an outcome among participants who actually had the outcome compared with those who did not.^17^ A precision-recall curve (PRC) was also included.^31^

We also provided a calibration plot^17^ for the ML model. Calibration is considered an important property of any prediction model; it reflects the extent to which predicted values align with observed values and is most often illustrated by a plot of observed vs predicted values.^13,17^ As well, we added a negative predictive value (NPV) vs predicted estimated risk plot to highlight the association between low estimated risk and a true-negative result (ie, those who did not experience the outcome).

From here, we reported 2 methods for health regulators to assess the clinical utility of the ML model. The first involved ranking our estimated risks, as others have done,^32,33^ by categorizing them into percentiles (eg, deciles) or keeping them in absolute numbers (eg, top 10 highest-risk dispensations). At each of these category cutoff points, we reported prediction performance metrics. These included positive likelihood ratios (LRs),^19,34^ true- and false-positive results, true- and false-negative results, and positive predictive values (PPVs; equivalent to posttest probability). These metrics were also reported on the actual thresholds of estimated risk outputted by the ML classifier. We carried out this analysis on all of the data from the validation set and measured these metrics at the end of 2019.

In the second method, we performed a decision curve analysis^35^ in which the net benefit of our ML model was compared against 2 alternatives—namely, intervening on all opioid dispensations or on none, using the entire range of probability threshold cutoff points. This comparison was done by using predicted probabilities from our ML model and comparing them against a probability threshold to aid a decision. This highlights the trade-off in prediction models where at various thresholds, the decision to intervene or not must be balanced with true and false detections. For example, it may be acceptable to miss several patients at high risk of opioid-related adverse outcomes to avoid many unnecessary interventions for those with low risk, which may have minimal impact on patients. Net benefit analysis can provide some information to health regulators to reconcile this matter.^36^ Decision curve analysis or net benefit simply presents another lens with which to evaluate ML prediction performance and/or utility in addition to discrimination, calibration, and the other commonly used metrics.

Thus, if a health regulator such as the CPSA is interested in intervening, for example, on the top 1 percentile of estimated risk or top 10 highest-risk predicted opioid dispensations, then method 1 could be considered. Alternatively, if they want to intervene on opioid dispensations above a certain estimated risk threshold, method 2 could be informative. Either way, the workload created by identifying high-risk dispensations is an important factor for the health regulator when applying this ML classifier; any interventions aided by ML prediction should only increase workload to a manageable extent.

Because we are using Alberta data, we simulated predictions to view the capabilities of our ML model if deployed into the CPSA workflow. These included predictions measured daily and weekly that progressively excluded participants once they were already flagged as high risk. Filtering out previously flagged patients represents a more realistic scenario for any health regulator, as it is not practical to repeatedly identify the same high-risk patients. For this simulation, previously flagged participants were excluded for the entire year while keeping in mind that a health regulator could exclude patients on a monthly or quarterly basis. For comparison, we reported the results of simulating and stratifying predictions using percentiles by not progressively excluding participants previously flagged as high risk. We also simulated the number of 30-day events per 100 daily dispenses stratified by percentiles of risk. Considering estimated risk thresholds and workload, we report how many high-risk dispensations the CPSA would have to consider based on threshold cutoff points. Finally, we briefly describe the general format of an electronic dashboard that health regulators like the CPSA could use in their opioid stewardship program.

We did not anticipate any missing data in our study because TPP Alberta PIN data involve a full capture of all information. All analyses were done using Python, version 3.6.8 (Python Software Foundation); SciKit-Learn, version 0.23.2 (SciKit-Learn Consortium)^37^; SHAP (Shapley Additive Explanations), version 0.35 (SHAP Developers)^38^; XGBoost, version 0.90 (XGBoost Developers)^16^; Pandas, version 1.0.5 (Pandas Development Team)^39^; and Stata/MP, version 15.1 (StataCorp LLC).

## Results

A total of 853 324 participants were included in this study, representing 6 181 025 opioid dispensations during 2018 to 2019; 46.4% of the participants were men and 53.6% were women, with a mean (SD) age of 49.1 (15.6) years for men and 51.0 (18.0) years for women. During the same time period, 145 016 composite outcome events (2.3%) occurred. There were 112 425 (77.5%) ED visits, 32 511 (22.4%) hospitalizations, and 80 (0.1%) deaths. Dispensations in 2018 and 2019 were the development and validation sets, respectively (eFigure 1 in Supplement 1). The validation set had 77 326 opioid dispensations with positive instances (2.6%) representing the pretest probability of the outcome, with a mean (SD) of approximately 8241 (2423) opioid dispensations per day (eFigure 3 in Supplement 1). Characteristics were comparable for those in the development and validation sets (eTable 3 in Supplement 1), while differences were noted among those who experienced the outcome and those who did not (eTable 4 in Supplement 1), as was expected.

**Figure 1.**
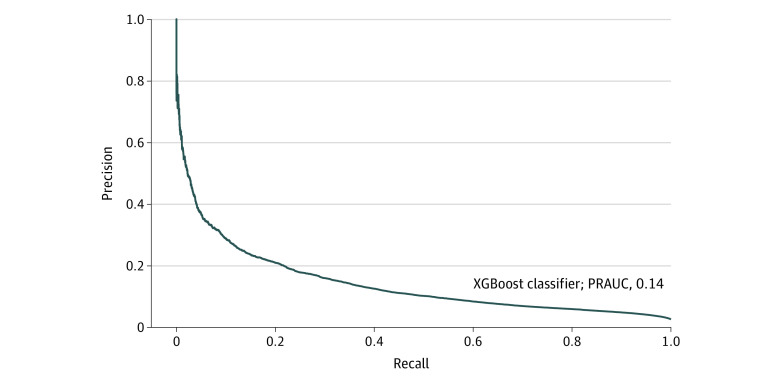
Precision-Recall Curve of Our XGBoost Classifier Using the 2019 Validation Data Our findings indicate the trade-off between precision (ie, positive predicted value) and recall (ie, sensitivity). PRAUC indicates precision-recall area under the curve.

Using the entire validation set, we estimated the AUROC for the XGBoost classifier to be 0.82 (95% CI, 0.81-0.82; eFigure 4 in Supplement 1) and an area under the PRC of 0.13. As is commonly seen with class-imbalanced outcomes (ie, rare outcomes), our findings indicate a PPV-sensitivity trade-off in which PPV decreases as sensitivity increases and vice versa (Figure 1). As for the calibration plot, the observed and estimated risks were not aligned and showed a consistent overestimation of risk, with a substantial fraction of instances predicted as low risk (eFigure 5 in Supplement 1). Low estimated risks were accompanied by fewer actual outcomes highlighting higher NPVs at lower estimated risks (eFigure 6 in Supplement 1).

After we ranked and grouped our estimated risks at the end of 2019, the categories with the highest risk predictions had the highest PPVs (and posttest probabilities) and positive LRs. The top 0.1 percentile of estimated risk had a positive LR of 28.7, which translated to a posttest probability of 43.1% compared with the pretest probability of 2.6% (Table 1). Similar results were observed with the top 10 high-risk dispensations reporting a PPV of 0.9 and a positive LR of 341 (Table 1). There was also an increase in positive LR and PPV as the threshold of estimated risk increased (eTable 5 in Supplement 1).

**Table 1.** XGBoost Prediction Metrics Measured at the End of 2019

Metric	Predicted probability threshold	TP	FP	FN	TN	PPV^a^	Sensitivity	Specificity	PLR
Top dispensations									
10	0.98	9	1	77 317	2 931 070	0.90	<0.001	1.00	341.1
50	0.98	46	4	77 280	2 931 067	0.92	0.001	1.00	435.9
100	0.98	79	21	77 247	2 931 050	0.79	0.001	1.00	142.6
500	0.97	250	250	77 076	2 930 821	0.50	0.003	1.00	37.9
1000	0.96	498	502	76 828	2 930 569	0.50	0.01	1.00	37.6
5000	0.94	2014	2986	75 312	2 928 082	0.40	0.03	1.00	25.5
10 000	0.92	3462	6538	73 864	2 924 533	0.35	0.04	1.00	20.1
50 000	0.86	10 779	39 221	66 547	2 891 850	0.22	0.14	0.99	10.4
100 000	0.81	17 238	82 762	60 088	2 848 309	0.17	0.22	0.97	7.9
Top percentile of estimated risk									
0.01	0.97	183	118	77 143	2 930 953	0.61	0.002	1.00	55.5
0.1	0.95	1295	1713	76 031	2 929 358	0.43	0.02	1.00	28.7
1	0.88	7559	22 525	69 767	2 908 546	0.25	0.10	0.99	12.7
5	0.78	22 175	128 245	55 151	2 802 826	0.15	0.29	0.96	6.6
10	0.71	32 182	268 658	45 144	2 662 413	0.11	0.43	0.91	4.5
25	0.57	51 526	700 573	25 800	2 230 498	0.07	0.67	0.76	2.8
50	0.35	69 659	1 434 539	7667	1 496 532	0.05	0.90	0.51	1.8
75	0.15	75 719	2 180 579	1607	750 492	0.03	0.98	0.26	1.3
90	0.07	76 968	2 630 589	358	300 482	0.03	1.00	0.10	1.1

Abbreviations: FN, false negative; FP, false positive; PLR, positive likelihood ratio; PPV, positive predictive value (posttest probability); TN, true negative; TP, true positive.

^a^ Compared with a pretest probability of 2.6% based on prevalence.

When we performed the decision-curve analysis across the entire range of threshold probabilities, the XGBoost classifier provided minimal improvements than if all or none of the participants were considered high risk (eFigure 7 in Supplement 1). For study participants with estimated risk probabilities across most of the range of thresholds, the net benefit was greatest if no interventions were performed at all and our XGBoost classifier did not add sufficient information to improve care. This was despite the high discrimination performance of the ML model.

**Figure 2.**
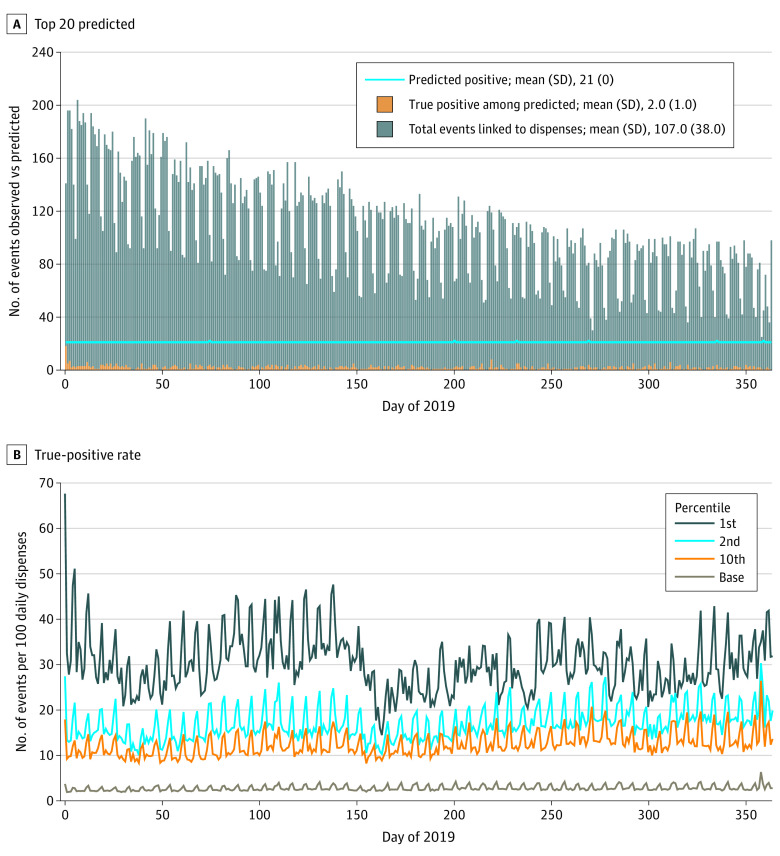
Simulation of Predicting the Top 20 Riskiest Opioid Dispensations Measured Daily by Progressively Excluding Participants Previously Flagged as High Risk A, Top 20 riskiest dispensations. The solid blue line (what was predicted) represents a workload that the College of Physicians and Surgeons of Alberta (CPSA) would have to consider. B, True-positive rate. The XGBoost classifier predicted daily risks in a simulation for the CPSA stratified by top percentile categories of risk. Base risk is 2.6% and represents the pretest probability.

In our simulations, the predictions classified weekly had higher positive LRs in both the highest-risk dispensations and percentiles of estimated risk compared with predictions classified daily. Measured weekly, we reported a positive LR of 20.55 in the top 20 dispensations, corresponding to a PPV (posttest probability) of 0.20. This is in comparison to daily measured predictions, which had a positive LR and a PPV of 6.38 and 0.09, respectively (Table 2). The same trend occurred when we assessed weekly vs daily predictions using top percentiles of estimated risk (eTable 6 in Supplement 1). By progressively excluding previously flagged participants and measuring the top 20 riskiest dispensations, the total number of 30-day events decreased as the year progressed (Figure 2A). When we assessed performance in the top percentiles of estimated risk and did not exclude previously flagged participants, positive LRs were higher (eTable 7 in Supplement 1) compared with the results of excluding previously flagged participants. Considering actual probability thresholds and workload, as the predicted probability threshold cutoff point increased, the number of flagged dispensations decreased for predictions measured daily and weekly (Table 3). Also, higher thresholds were connected to a higher positive LR, with weekly measurements being more informative than daily ones (Table 3).

**Table 2.** Prediction Metrics Simulated Using Daily and Weekly Measurements Stratified by Top Dispensations Using 2019 Data^a^

Top dispensations	Predicted probability threshold	TP	FN	FP	TN	PPV^b^	NPV	Sensitivity	Specificity	PLR
Measured daily										
10	0.83	1	127	10	7506	0.12	0.98	0.01	1.00	8.00
20	0.78	2	105	19	7108	0.09	0.99	0.02	1.00	6.38
50	0.67	3	65	49	6167	0.06	0.99	0.04	0.99	5.63
100	0.54	4	41	99	5187	0.04	0.99	0.09	0.98	4.74
200	0.40	5	24	203	4204	0.02	0.99	0.17	0.95	3.76
500	0.22	6	9	627	2517	0.01	1.00	0.41	0.80	2.07
1000	0.09	7	3	1185	929	0.01	1.00	0.69	0.44	1.24
Measured weekly^c^										
10	0.92	3	510	8	39 838	0.24	0.99	0.01	1.00	24.66
20	0.90	4	488	17	39 701	0.20	0.99	0.01	1.00	20.55
50	0.86	8	444	43	39 285	0.15	0.99	0.02	1.00	15.25
100	0.81	11	395	90	38 627	0.11	0.99	0.03	1.00	11.64
200	0.74	16	333	186	37 341	0.08	0.99	0.04	1.00	9.06
500	0.61	25	218	496	33 860	0.05	0.99	0.10	0.99	7.20
1000	0.46	31	145	1018	29 377	0.03	1.00	0.18	0.97	5.24

Abbreviations: FN, false negative; FP, false positive; PLR, positive likelihood ratio; NPV, negative predictive value; PPV, positive predictive value (posttest probability); TN, true negative; TP, true positive.

^a^ Analysis was based on mean daily and weekly values to prevent daily and weekly fluctuations of dispensations. Participants were progressively excluded for 1 year if previously flagged as high risk.

^b^ Compared with pretest probability of 2.6% based on prevalence.

^c^ The highest predicted probability of the week was used.

**Table 3.** Prediction Metrics Simulated Using Daily and Weekly Measurements Stratified by Absolute Probability Thresholds Using 2019 Data^a^

Predicted probability threshold	No. of flagged dispensations	TP	FN	FP	TN	PPV^b^	NPV	Sensitivity	Specificity	PLR
Measured daily										
0	1457	7	0	1450	0	0.00	NA	1.00	0.00	1.00
0.1	1009	7	1	1002	811	0.01	1.00	0.90	0.45	1.63
0.2	569	6	3	563	1825	0.01	1.00	0.69	0.76	2.93
0.3	316	5	6	311	2578	0.02	1.00	0.49	0.89	4.52
0.4	217	5	9	212	3126	0.02	1.00	0.35	0.94	5.54
0.5	150	4	13	146	3692	0.03	1.00	0.25	0.96	6.51
0.6	98	4	23	94	4479	0.04	0.99	0.14	0.98	6.76
0.7	53	3	52	51	5676	0.05	0.99	0.05	0.99	5.82
0.8	22	2	91	21	6825	0.08	0.99	0.02	1.00	6.47
0.9	4	1	154	3	7757	0.15	0.98	0.00	1.00	8.96
1	0	0	212	0	8029	NA	0.97	0.00	1.00	NA
Measured weekly^c^										
0	10 198	50	0	10148	0	0.00	NA	1.00	0.00	1.00
0.1	7060	48	5	7013	5529	0.01	1.00	0.90	0.44	1.62
0.2	3984	42	18	3941	12 411	0.01	1.00	0.70	0.76	2.91
0.3	2212	37	36	2175	17 483	0.02	1.00	0.51	0.89	4.58
0.4	1516	33	54	1483	21 083	0.02	1.00	0.38	0.93	5.79
0.5	1051	31	79	1020	24 570	0.03	1.00	0.28	0.96	6.97
0.6	683	26	123	656	28 539	0.04	1.00	0.18	0.98	7.82
0.7	374	20	209	355	33 097	0.05	0.99	0.09	0.99	8.10
0.8	157	13	319	144	36 904	0.08	0.99	0.04	1.00	9.77
0.9	29	4	465	24	39 418	0.15	0.99	0.01	1.00	15.18
1	0	0	558	0	39 988	NA	0.99	0.00	1.00	NA

Abbreviations: FN, false negative; FP, false positive; PLR, positive likelihood ratio; NA, not available; PPV, positive predictive value (posttest probability); TN, true negative; TP, true positive.

^a^ Participants were progressively excluded for 1 year if previously flagged as high risk. Analysis was based on mean daily and weekly values to prevent daily and weekly fluctuations of dispensations. The number of flagged dispensations represents a potential College of Physicians and Surgeons of Alberta workload.

^b^ Compared with pretest probability of 2.6% based on prevalence.

^c^ The highest predicted probability of the week was used.

When we simulated based on top percentiles of estimated risk and events per 100 daily dispensations, the highest 1 percentile of estimated risk had higher event rates than the lower estimated risk percentiles, including the baseline risk (pretest probability of approximately 2.6%; Figure 2B).

We included the outputs from our ML model within an electronic dashboard that allows the CPSA to identify patients at high risk for a 30-day event and decide what, if any, interventions to initiate. This view of the data allows the CPSA to filter an aggregated patient list based on ML risk prediction scores, age, and characteristics of medication use (eg, oral morphine equivalents, benzodiazepine dispensations). Full dispensation profiles are also available. To help with use of the dashboards, we also included a page with general use instructions and a list of all features along with a brief description for each. It is also possible for users to search for a particular patient even if they are not among individuals with the highest estimated risk.

## Discussion

In this prognostic study, we created an XGBoost ML classifier for PDMPs to assess risk from prescribed opioids using the CPSA as an example of a health regulator. We presented 2 analytic options for health regulators to assess and implement ML decision support into an opioid stewardship workflow: namely, acting on the highest ranked predictions or on probability threshold cutoffs. Discrimination, calibration, and net benefit analysis are important aspects in determining the clinical utility of a prediction model.^35^ Although our model had high discrimination performance, it did not calibrate well.

Intervention based on a probability threshold or on ranked predictions is a decision for health regulators to make depending on their use-case scenarios. There are challenges with assessing our ML model with net benefit analysis. When it comes to adverse outcomes of prescribed opioids, there are no generally accepted or guideline-recommended probability risk thresholds on which to intervene unless a health regulator decides to act on an arbitrary threshold, which may be the case. Our net benefit analysis suggests that using probability thresholds, arbitrary or not, to guide interventions may not lead to an informative decision aid. This could change if health regulators managing PDMPs had access to other types of data.

There may be some value in ranking predictions, as some positive LRs led to conclusive changes from pretest to posttest probabilities,^19^ with weekly predictions being more informative than daily ones. Using this option of ranking predictions, health regulators could implement ML prediction as a decision aid to potentially intervene on high-risk opioid dispensations. The electronic dashboard we described could assist PDMPs by identifying high-risk patients and can be adjusted according to acceptable workloads by varying the number of identified high-risk instances. Regulators could also progressively exclude previously flagged patients yearly (as we did), quarterly, or at another frequency based on workload capacity.

Health regulators such as the CPSA have a mandate to ensure high-quality medical care while protecting the public, and the goals of interventions should include proactively reducing patient risk. As such, our findings suggest that ML prediction could help inform interventions like provider notifications and self-directed and/or audit-based feedback.

A challenge commonly encountered in prediction models is the PPV sensitivity, or precision-recall, trade-off. This occurs in studies with rare outcomes like ours. Indeed, as the threshold varies in our ML model, the trade-off between PPV and sensitivity becomes apparent, with PPV increasing while sensitivity decreases and vice versa. End users, such as health regulators administering PDMPs, must identify their priorities and reconcile this issue with acceptable workloads, as achieving both high PPV and sensitivity is difficult. For example, if a health regulator chooses an ML model with high sensitivity, most high-risk patients will be detected, but many interventions will be applied to those with actual low risk and perhaps create unnecessarily high workloads. On the other hand, if a model with high PPV is preferred (to avoid unnecessary interventions), many patients at risk may remain undetected.

### Limitations

This study has limitations, which are mainly due to data issues. Our training data set does not account for the risk associated with nonprescription opioid use. The CPSA’s limited data access did not allow for any exclusion criteria in our study population; thus, we were not able to exclude any participants based on comorbidity or other (eg, cancer, palliative care) histories. Miscalibration and uninformative net benefit analysis could partly be explained by the limited data available to the CPSA. From previous work, it is well known that having more data could better leverage ML prediction capabilities,^11,40^ thus making the case to increase data access and permissions for the CPSA and regulators in general. Indeed, this issue could be mitigated by allowing the CPSA increased access to other administrative data sets storing social factors and comorbidity data for training ML models. Our ML classifier was trained only on Alberta data, which may not be generalizable to other jurisdictions. However, the ML process makes it easier to train models using population-specific data.

To date, we are unaware of other Canadian jurisdictions that have implemented or studied ML prediction to aid PDMPs in reducing risk from opioids. Although our ML model was trained on the limited data available to the CPSA, its discrimination performance was comparable to results from our previous work and that of others.^10^ Furthermore, prediction performance using ranked predictions was comparable to that in our previous work, in which we had access to more types of training data than the CPSA.

Although we found no other published work describing ML-assisted PDMPs, others have studied ML prediction and opioid outcomes in a more general setting. We identified 1 study^10^ in which the authors used dispensation-level data, like we did, to construct ML models to estimate opioid-related adverse outcomes using data sets much more extensive than ours. Most likely due to the limited nature of our data, their ML models reported higher discrimination performance than ours (0.82 vs 0.90). Like that study, our ML model had better prediction performance in the higher-risk group. Indeed, access to more diverse data could improve ML prediction performance. Nevertheless, our study suggests some implementation potential with limited data.

## Conclusions

This study considered a particular regulator’s perspective with respect to ML prediction performance, clinical utility, and workload when predicting opioid-related outcomes. This approach can be applied to other health regulators with similar opioid stewardship mandates. Training on data representing specific populations and regular retraining to improve prediction performance over time are among the benefits of the ML process. Our XGBoost classifier gives health regulators options for interventions based on workload capacity; any potential interventions on opioid dispensations can be identified at the patient level, in which opioid use could be further assessed. Improved data access could improve prediction performance, leading to more effective opioid stewardship. Future work should consider the nature of interventions and whether those interventions in fact lead to observed decreases in adverse outcomes.

## Notes

Supplement 1. **eFigure 1.** Study Participant Flow Diagram **eTable 1.** Anatomical Therapeutic Chemical Classification of Opioid Molecules Used for This Study and Candidate Predictors Used to Develop the XGBoost Classifier **eTable 2.** Diagnostic Codes Used to Identify the Defined Study Outcome From Emergency Department Visit, Hospitalization, and Death Data **eTable 3.** Characteristics of the Study Participants (N = 853 324) in the Development and Validation Sets **eTable 4.** Characteristics of the Study Participants (N = 853 324) According to Outcome Status **eTable 5.** XGBoost Prediction Performance Metrics Based on Threshold of Estimated Risk Measured at the End of 2019 **eTable 6.** Prediction Metrics Simulated Using Daily and Weekly Measurements Stratified by Percentiles of Estimated Risk Using 2019 Data **eTable 7.** Prediction Metrics Simulated Using Daily and Weekly Measurements Stratified by Top Percentiles of Estimated Risk Using 2019 Data **eFigure 2.** Schematic of Study Design and Feature Generation **eFigure 3.** Number of Opioid Dispensations per Day During 2019 (Mean 8241 Dispensations per Day) and the Number of Dispensations That Resulted in Our Defined Outcome (Mean 212 or Approximately 2.6% of Dispensations That Led to an Event) **eFigure 4.** Discrimination Performance of Our XGBoost Classifier Using the Entire 2019 Validation Data Set **eFigure 5.** Calibration Plot of the XGBoost Classifier Using 2019 Data **eFigure 6.** Negative Predicted Value vs Predicted Probability Using the 2019 Validation Set **eFigure 7.** Decision-Curve Analysis **eAppendix.** Feature List

Supplement 2.
Data Sharing Statement
