# Supplementary material for: Development and Validation of a Machine Learning Model to Estimate Risk of Adverse Outcomes Within 30 Days of Opioid Dispensation
Source: JAMA Netw Open. 2022 Dec 27;5(12):e2248559. doi: 10.1001/jamanetworkopen.2022.48559 (PMC9857580; doi:10.1001/jamanetworkopen.2022.48559)
Supplement: Supplement 1. — eFigure 1. Study Participant Flow Diagram eTable 1. Anatomical Therapeutic Chemical Classification of Opioid Molecules Used for This Study and Candidate Predictors Used to Develop the XGBoost Classifier eTable 2. Diagnostic Codes Used to Identify the Defined Study Outcome From Emergency Department Visit, Hospitalization, and Death Data eTable 3. Characteristics of the Study Participants (N = 853 324) in the Development and Validation Sets eTable 4. Characteristics of the Study Participants (N = 853 324) According to Outcome Status eTable 5. XGBoost Prediction Performance Metrics Based on Threshold of Estimated Risk Measured at the End of 2019 eTable 6. Prediction Metrics Simulated Using Daily and Weekly Measurements Stratified by Percentiles of Estimated Risk Using 2019 Data eTable 7. Prediction Metrics Simulated Using Daily and Weekly Measurements Stratified by Top Percentiles of Estimated Risk Using 2019 Data eFigure 2. Schematic of Study Design and Feature Generation eFigure 3. Number of Opioid Dispensations per Day During 2019 (Mean 8241 Dispensations per Day) and the Number of Dispensations That Resulted in Our Defined Outcome (Mean 212 or Approximately 2.6% of Dispensations That Led to an Event) eFigure 4. Discrimination Performance of Our XGBoost Classifier Using the Entire 2019 Validation Data Set eFigure 5. Calibration Plot of the XGBoost Classifier Using 2019 Data eFigure 6. Negative Predicted Value vs Predicted Probability Using the 2019 Validation Set eFigure 7. Decision-Curve Analysis eAppendix. Feature List [file jamanetwopen-e2248559-s001.pdf]

Supplementary Online Content

Sharma V, Kulkarni V, Jess E, et al. Development and validation of a machine learning model to estimate risk of adverse outcomes within 30 days of opioid dispensation. *JAMA Netw Open*. 2022;5(12):e2248559. doi:10.1001/jamanetworkopen.2022.48559

eFigure 1. Study Participant Flow Diagram
eTable 1. Anatomical Therapeutic Chemical Classification of Opioid Molecules Used for This Study and Candidate Predictors Used to Develop the XGBoost Classifier
eTable 2. Diagnostic Codes Used to Identify the Defined Study Outcome From Emergency Department Visit, Hospitalization, and Death Data
eTable 3. Characteristics of the Study Participants (N = 853 324) in the Development and Validation Sets
eTable 4. Characteristics of the Study Participants (N = 853 324) According to Outcome Status
eTable 5. XGBoost Prediction Performance Metrics Based on Threshold of Estimated Risk Measured at the End of 2019
eTable 6. Prediction Metrics Simulated Using Daily and Weekly Measurements Stratified by Percentiles of Estimated Risk Using 2019 Data
eTable 7. Prediction Metrics Simulated Using Daily and Weekly Measurements Stratified by Top Percentiles of Estimated Risk Using 2019 Data
eFigure 2. Schematic of Study Design and Feature Generation
eFigure 3. Number of Opioid Dispensations per Day During 2019 (Mean 8241 Dispensations per Day) and the Number of Dispensations That Resulted in Our Defined Outcome (Mean 212 or Approximately 2.6% of Dispensations That Led to an Event)
eFigure 4. Discrimination Performance of Our XGBoost Classifier Using the Entire 2019 Validation Data Set
eFigure 5. Calibration Plot of the XGBoost Classifier Using 2019 Data
eFigure 6. Negative Predicted Value vs Predicted Probability Using the 2019 Validation Set
eFigure 7. Decision-Curve Analysis
eAppendix. Feature List

This supplementary material has been provided by the authors to give readers additional information about their work.

© 2022 Sharma V et al. *JAMA Network Open*.

eFigure 1. Study Participant Flow Diagram

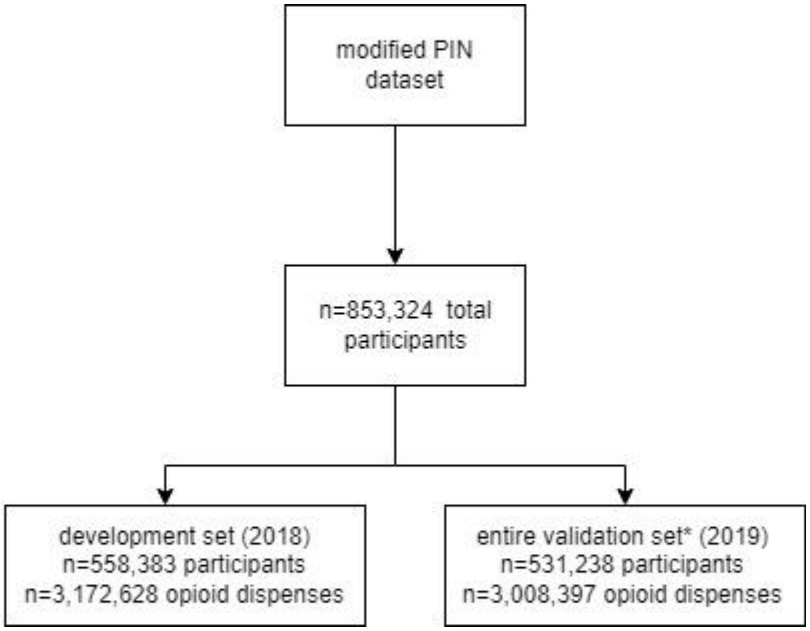

*Participants could be in both the development and validation set; the analysis was conducted on this validation set

Note: PIN=Pharmaceutical Information Network

© 2022 Sharma V et al. *JAMA Network Open*.

eTable 1. Anatomical Therapeutic Chemical Classification of Opioid Molecules Used for This Study and Candidate Predictors Used to Develop the XGBoost Classifier

Category (data source)	Description
ATC codes used to identify opioids from modified PIN data	N01AH01, N01AH03, N01AH06, N07BC01, N07BC02, N07BC51, R05DA03, R05DA04, R05DA09, R05DA20, N02A
Opioid molecules used in this study (modified PIN)	alfentanil, butorphanol, codeine, diamorphine, fentanyl, hydrocodone, hydromorphone, meperidine, morphine, oxycodone, oxymorphone, pentazocine, sufentanil, tapentadol, tramadol
Demographic information (modified PIN)	age, sex, postal codes, mean income
Drug utilization history (modified PIN)	drug dispenses in past 30 days using ATC codes, oral morphine equivalents, concurrent use with benzodiazepines defined as at least 7 days of cumulative concurrent use in the 30 days prior to dispensation, number of dispensations and unique molecules of opioids and benzodiazepines
Healthcare utilization (modified PIN)	Number of opioid prescribers and pharmacies

Note: ATC- Anatomical Therapeutic Chemical classification (https://www.whocc.no/atc_ddd_index); modified PIN- Pharmaceutical Information Network data modified to align with data access granted to the College of Physicians and Surgeons of Alberta.

eTable 2. Diagnostic Codes Used to Identify the Defined Study Outcome From Emergency Department Visit, Hospitalization, and Death Data

ICD 10-CA*	Condition
**T40.x**	Poisoning by, adverse effect of and underdosing of narcotics and psychodysleptics
**F55.x**	Abuse of non-psychoactive substances
**F11.x - F19.x**	Mental and behavioral disorders due to psychoactive substance use

***** International Statistical Classification of Diseases and Related Health Problems, 10th Revision, Canada

© 2022 Sharma V et al. *JAMA Network Open*.

eTable 3. Characteristics of the Study Participants (N = 853 324) in the Development and Validation Sets

Characteristic	Number in development set *	Percent	Number in validation set*	Percent
**Number of participants**	558,383	100.0	531,238	100.0
**Age:**				
Mean (SD)	50.7 (17.6)	(--)	51.0 (17.7)	(--)
18-45	226,648	40.6	213,456	40.2
45-65	212,760	38.1	200,816	37.8
>65	118,975	21.3	116,966	22.00
**Male**	265,954	54.0	243,639	45.9
**Female**	301,429	46.0	287,599	54.1
**Rural**	88,783	15.9	83,713	15.8
**Urban**	469,600	84.1	447,525	84.2
**Income:**				
<75k	83,010	14.9	77,901	14.7
75k-100k	286,509	51.3	270,397	50.9
100k-171k	175,837	31.5	171,038	32.2
>171k	13,027	2.3	11,902	2.2
**Number of unique physicians visited in the 30-days prior to opioid dispensation:**				
1 to 3	532,546	95.4	503,745	94.8
>3	25,837	4.6	27,493	5.2

*Unless otherwise indicated

Note: p<0.001 for all comparisons due to large sample sizes except rural/urban (p=0.04); participants can be in either or both categories; number of unique physicians was never 0 because each line in our data set represented an opioid dispensation which inherently requires at least one physician prescriber.

© 2022 Sharma V et al. *JAMA Network Open*.

eTable 4. Characteristics of the Study Participants (N = 853 324) According to Outcome Status

Characteristic	Number with event*	Percent	Number without event*	Percent
**Number of participants**	14,916	100.0	852,065	100.0
**Age:**				
Mean (SD)	45.8 (15.2)	(--)	49.8 (17.8)	(--)
18-45	7,689	51.5	367,777	43.2
45-65	5,677	38.1	310,897	36.5
>65	1,550	10.4	173,391	20.3
**Male**	8,191	54.9	394,935	46.4
**Female**	6,725	45.1	457,130	53.6
**Rural**	3,536	23.7	128,621	15.1
**Urban**	11,380	76.3	723,444	84.9
**Income:**				
<75k	4,104	27.5	120,805	14.2
75k-100k	8,069	54.1	431,444	50.6
100k-171k	2,492	16.7	279,699	32.8
>171k	251	1.7	20,117	2.4
**Number of unique physicians visited in the 30-days prior to opioid dispensation:**				
1 to 3	10,231	68.6	823,167	96.6
>3	4,685	31.4	28,898	3.4

*Unless otherwise indicated.

Note: p<0.001 for all comparisons between event and no event; participants can be in either or both categories because some instances lead to an event while others do not within the same participant; number of unique physicians was never 0 because each line in our data set represented an opioid dispensation which inherently requires at least one physician prescriber.

© 2022 Sharma V et al. *JAMA Network Open*.

eTable 5. XGBoost Prediction Performance Metrics Based on Threshold of Estimated Risk Measured at the End of 2019

Threshold of predicted risk	TP	FN	FP	TN	PPV*	Se	Sp	LR+
**0**	77,326	0	2,931,071	0	0.026	1.000	0.000	1.00
**0.1**	76,623	703	2,440,890	490,181	0.030	0.991	0.167	1.19
**0.2**	74,568	2,758	1,927,842	1,003,229	0.037	0.964	0.342	1.47
**0.3**	71,536	5,790	1,573,273	1,357,798	0.043	0.925	0.463	1.72
**0.4**	66,987	10,339	1,288,735	1,642,336	0.049	0.866	0.560	1.97
**0.5**	58,785	18,541	939,305	1,991,766	0.059	0.760	0.680	2.37
**0.6**	47,742	29,584	598,938	2,332,133	0.074	0.617	0.796	3.02
**0.7**	33,725	43,601	293,576	2,637,495	0.103	0.436	0.900	4.35
**0.8**	19,145	58,181	98,486	2,832,585	0.163	0.248	0.966	7.37
**0.9**	5,385	71,941	13,419	2,917,652	0.286	0.070	0.995	15.21
**0.99**	0	77,326	0	2,931,071	(--)	0.000	1.000	(--)

TP: true positives; FP: false positives; FN: false negatives; TN: true negatives; Se: sensitivity; Sp: specificity; LR+: positive likelihood ratio; PPV: positive predictive value, post-test probability
*Compared with pre-test probability of 2.6% based on prevalence.

© 2022 Sharma V et al. *JAMA Network Open*.

eTable 6. Prediction Metrics Simulated Using Daily and Weekly Measurements Stratified by Percentiles of Estimated Risk Using 2019 Data

Participants were progressively excluded for 1 year if previously flagged as high risk.

	Top Percentile of predicted risk	Threshold	TP	FN	FP	TN	PPV*	NPV	Se	Sp	LR+
**Measured daily**	**0.1**	0.86	1	141	6	7664	0.14	0.98	0.01	1.00	9.14
	**1**	0.66	3	63	49	6153	0.06	0.99	0.04	0.99	5.68
	**5**	0.41	5	21	173	4181	0.03	0.99	0.18	0.96	4.66
	**10**	0.31	5	11	296	3349	0.02	1.00	0.32	0.92	3.95
	**25**	0.20	6	4	643	2058	0.01	1.00	0.60	0.76	2.52
	**50**	0.11	7	1	982	1055	0.01	1.00	0.84	0.52	1.73
	**75**	0.07	7	0	1243	440	0.01	1.00	0.96	0.26	1.30
	**90**	0.04	7	0	1370	157	0.01	1.00	0.99	0.10	1.10
**Measured weekly****	**0.1**	0.89	5	474	24	39576	0.18	0.99	0.01	1.00	18.02
	**1**	0.71	18	299	240	36518	0.07	0.99	0.06	0.99	8.60
	**5**	0.46	31	128	992	28623	0.03	1.00	0.20	0.97	5.89
	**10**	0.34	36	77	1726	23802	0.02	1.00	0.32	0.93	4.74
	**25**	0.21	44	29	4265	14931	0.01	1.00	0.60	0.78	2.70
	**50**	0.12	48	9	6657	7815	0.01	1.00	0.84	0.54	1.82
	**75**	0.07	49	2	8636	3233	0.01	1.00	0.96	0.27	1.32
	**90**	0.04	49	1	9567	1155	0.01	1.00	0.99	0.11	1.11

Note: Based on average daily and weekly values to prevent daily and weekly fluctuations of dispenses.

Threshold: predicted probability threshold; TP: true positives; FP: false positives; FN: false negatives; TN: true negatives; Se: sensitivity; Sp: specificity; LR+: positive likelihood ratio; PPV: positive predictive value (post-test probability)

© 2022 Sharma V et al. *JAMA Network Open*.

*Compared with pre-test probability of 2.6% based on prevalence.
**The highest predicted probability of the week was used.

© 2022 Sharma V et al. *JAMA Network Open*.

eTable 7. Prediction Metrics Simulated Using Daily and Weekly Measurements Stratified by Top Percentiles of Estimated Risk Using 2019 Data

Participants were NOT progressively excluded if previously flagged as high risk.

	Top Percentile of predicted risk	Threshold	TP	FN	FP	TN	PPV*	NPV	Se	Sp	LR+
Measured daily	0.1	0.95	4	208	4	8025	0.47	0.97	0.02	1.00	33.78
	1	0.89	19	193	50	7979	0.28	0.98	0.09	0.99	14.38
	5	0.79	56	156	286	7743	0.16	0.98	0.26	0.96	7.44
	10	0.73	82	130	603	7426	0.12	0.98	0.39	0.92	5.13
	25	0.6	133	79	1633	6396	0.08	0.99	0.63	0.80	3.09
	50	0.39	187	25	3724	4305	0.05	0.99	0.88	0.54	1.90
	75	0.18	206	5	5748	2281	0.03	1.00	0.97	0.28	1.36
	90	0.09	210	1	7053	976	0.03	1.00	0.99	0.12	1.13
Measured weekly**	0.1	0.93	11	547	19	39969	0.36	0.99	0.02	1.00	41.03
	1	0.85	56	502	232	39756	0.19	0.99	0.10	0.99	17.23
	5	0.74	149	409	1294	38693	0.10	0.99	0.27	0.97	8.24
	10	0.66	223	335	2704	37283	0.08	0.99	0.40	0.93	5.90
	25	0.5	370	188	7579	32409	0.05	0.99	0.66	0.81	3.50
	50	0.29	483	75	16136	23851	0.03	1.00	0.87	0.60	2.15
	75	0.14	537	21	26759	13229	0.02	1.00	0.96	0.33	1.44
	90	0.08	553	5	34261	5727	0.02	1.00	0.99	0.14	1.16

Note: Based on average daily and weekly values to prevent daily and weekly fluctuations of dispenses.

Threshold: predicted probability threshold; TP: true positives; FP: false positives; FN: false negatives; TN: true negatives; Se: sensitivity; Sp: specificity; LR+: positive likelihood ratio; PPV: positive predictive value (post-test probability)

*Compared with pre-test probability of 2.6% based on prevalence.

**The highest predicted probability of the week was used.

© 2022 Sharma V et al. *JAMA Network Open*.

eFigure 2. Schematic of Study Design and Feature Generation

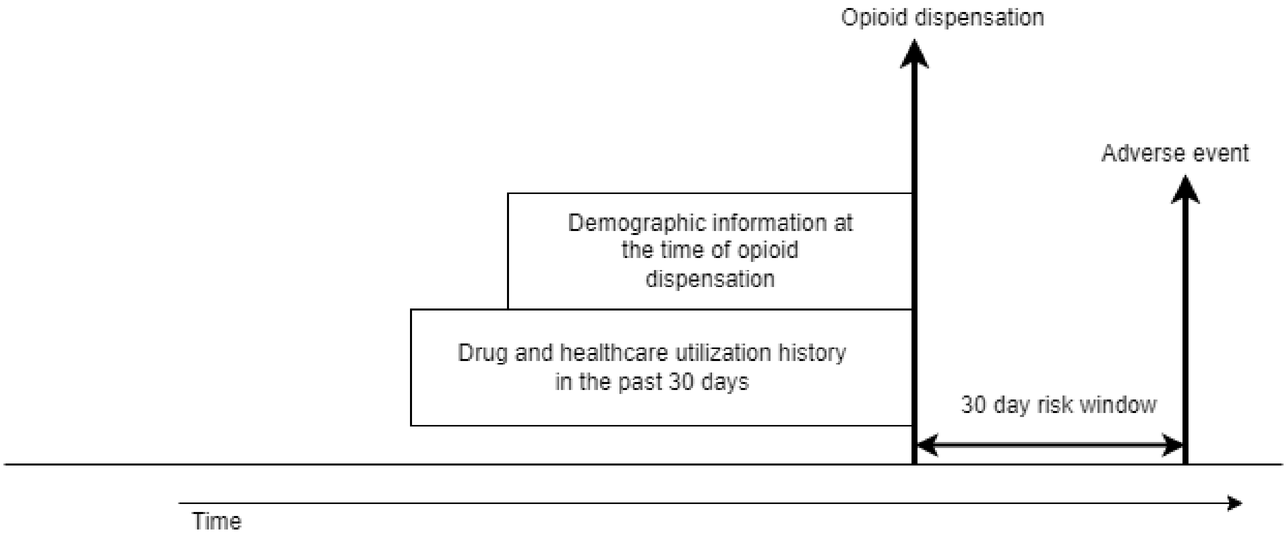

© 2022 Sharma V et al. *JAMA Network Open*.

eFigure 3. Number of Opioid Dispensations per Day During 2019 (Mean 8241 Dispensations per Day) and the Number of Dispensations That Resulted in Our Defined Outcome (Mean 212 or Approximately 2.6% of Dispensations That Led to an Event)

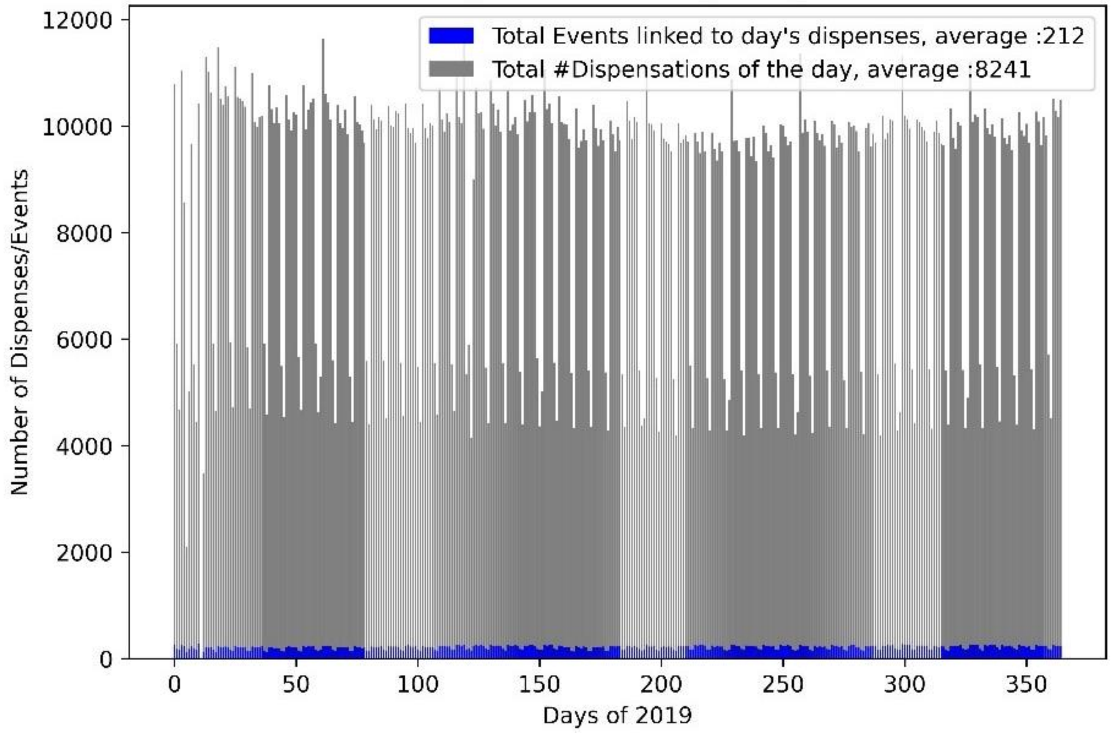

eFigure 4. Discrimination Performance of Our XGBoost Classifier Using the Entire 2019 Validation Data Set

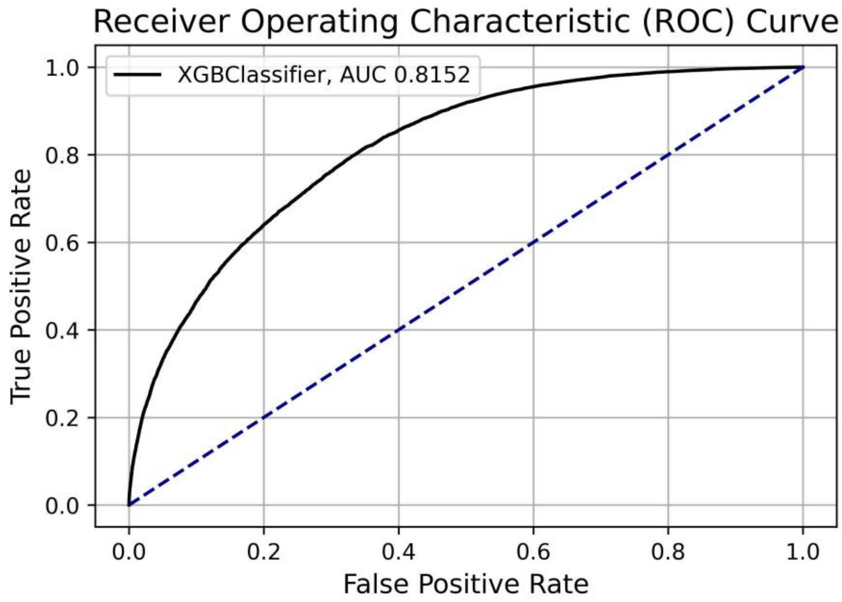

© 2022 Sharma V et al. *JAMA Network Open*.

eFigure 5. Calibration Plot of the XGBoost Classifier Using 2019 Data

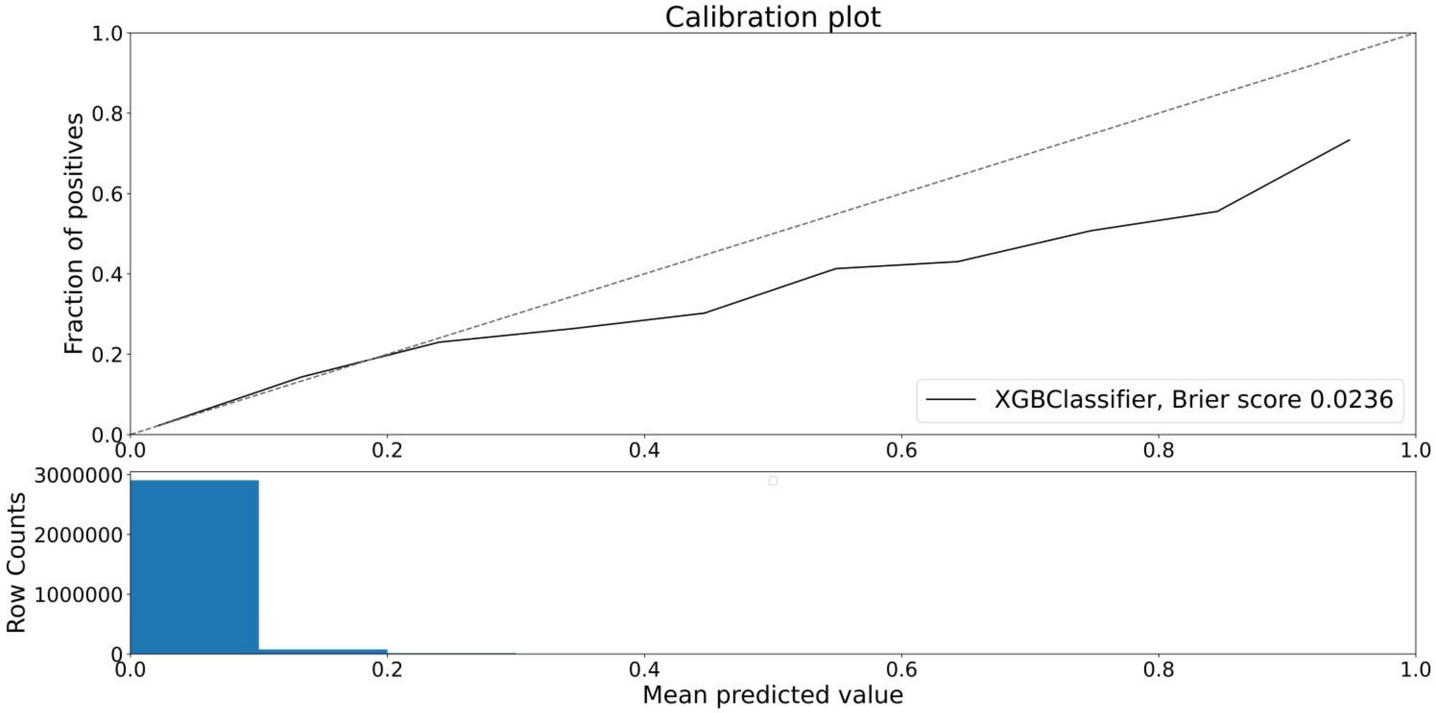

© 2022 Sharma V et al. *JAMA Network Open*.

eFigure 6. Negative Predicted Value vs Predicted Probability Using the 2019 Validation Set

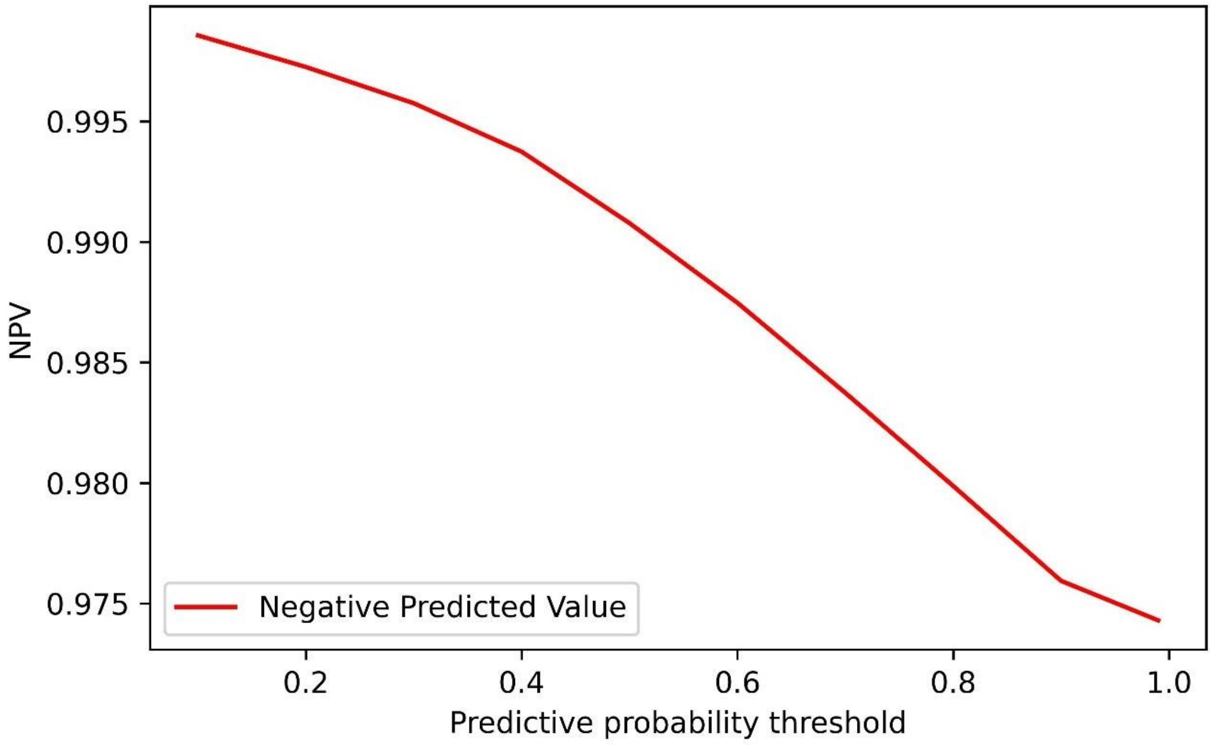

NPV: negative predicted value

eFigure 7. Decision-Curve Analysis

Across most of the range of threshold probabilities, the XGBoost classifier had a lower net benefit than if none of the opioid dispenses were intervened on. Thus, acting on predicted probability thresholds for interventions may not be informative nor appropriate.

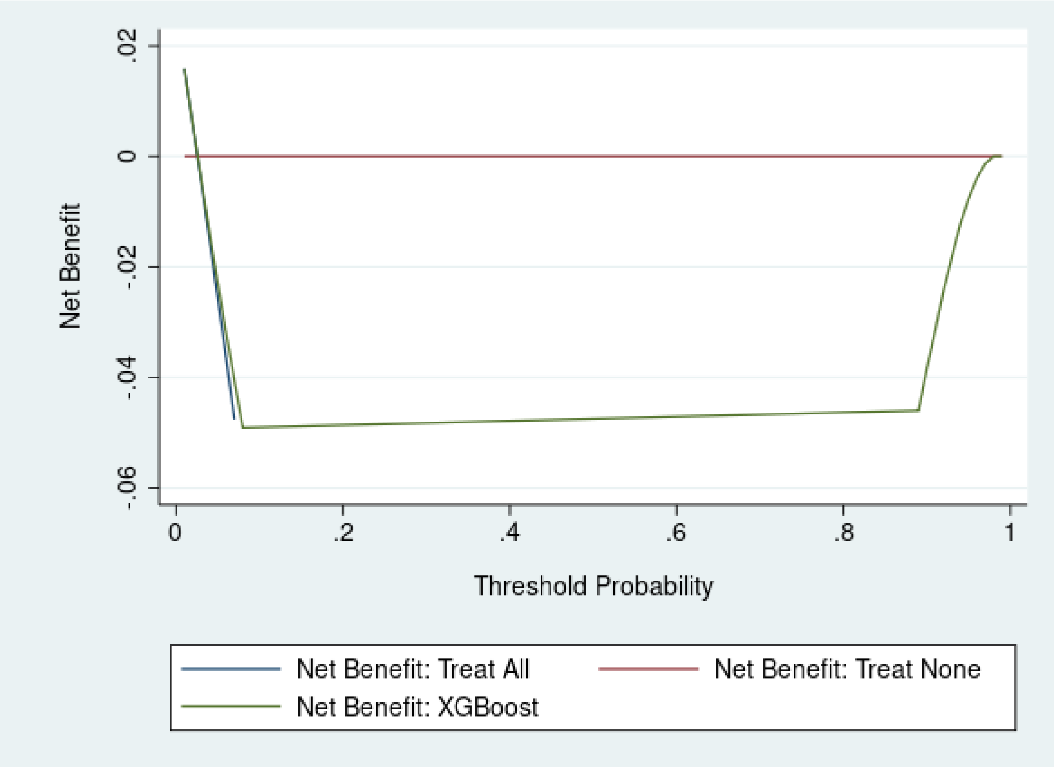

© 2022 Sharma V et al. *JAMA Network Open*.

eAppendix. Feature List

Category	Feature
**Demographic Information**	Age, sex, income, rural/urban status
**Level 3 ATC code (drug utilization history) ***	'N01A-ANESTHETICS, GENERAL', 'N03A-ANTIEPILEPTICS', 'N07B-DRUGS USED IN ADDICTIVE DISORDERS', 'N05B-ANXIOLYTICS', 'N02A-OPIOIDS', 'N05C-HYPNOTICS AND SEDATIVES', 'R05D-COUGH SUPPRESSANTS, EXCLUDING COMBINATIONS WITH EXPECTORANTS', 'M01A-ANTIINFLAMMATORY AND ANTIRHEUMATIC PRODUCTS, NON-STEROIDS', 'R06A-ANTIHISTAMINES FOR SYSTEMIC USE', 'J01C-BETA-LACTAM ANTIBACTERIALS, PENICILLINS', 'N06A-ANTIDEPRESSANTS', 'M03B-MUSCLE RELAXANTS, CENTRALLY ACTING AGENTS', 'N06B-PSYCHOSTIMULANTS, AGENTS USED FOR ADHD AND NOOTROPICS', 'A02B-DRUGS FOR PEPTIC ULCER AND GASTRO-OESOPHAGEAL REFLUX DISEASE (GERD)', 'J01F-MACROLIDES, LINCOSAMIDES AND STREPTOGRAMINS', 'N02B-OTHER ANALGESICS AND ANTIPYRETICS', 'N05A-ANTIPSYCHOTICS', 'J01A-TETRACYCLINES', 'G04B-OTHER UROLOGICALS, INCLUDING ANTISPASMODICS', 'A03F-PROPULSIVES', 'R03A-ADRENERGICS, INHALANTS', 'H02A-CORTICOSTEROIDS FOR SYSTEMIC USE, PLAIN', 'C03C-HIGH-CELING DIURETICS', 'D10B-ANTI-ACNE PREPARATIONS FOR SYSTEMIC USE', 'S02C-CORTICOSTEROIDS AND ANTIIFECTIVES IN COMBINATION', 'G03B-ANDROGENS', 'D06A-ANTIBIOTICS FOR TOPICAL USE', 'J01E-SULFONAMIDES AND TRIMETHOPRIM', 'A06A-LAXATIVES', 'S01A-ANTIINFECTIVES', 'A04A-ANTIEMETICS AND ANTINAUSEANTS', 'J01D-OTHER BETA-LACTAM ANTIBACTERIALS', 'C09A-ANGIOTENSIN-CONVERTING ENZYME (ACE) INHIBITORS, PLAIN', 'J01M-QUINOLONE ANTIBACTERIALS', 'L01B-ANTIMETABOLITES', 'J01X-OTHER ANTIBACTERIALS', 'B03X-OTHER ANTIANEMIC PREPARATIONS', 'A07A-INTESTINAL ANTIINFECTIVES', 'G01A-ANTI-INFECTIVES/ANTISEPTICS, EXCLUDING COMBINATIONS WITH CORTICOSTEROIDS', 'D07C-CORTICOSTEROIDS, COMBINATIONS WITH ANTIBIOTICS', 'A07D-ANTIPROPULSIVES', 'C05A-ANTIHEMORRHOIDALS FOR TOPICAL USE', 'N02C-ANTIMIGRAINE PREPARATIONS', 'R05F-COUGH SUPPRESSANTS AND EXPECTORANTS, COMBINATIONS', 'P01A-AGENTS AGAINST AMOEBIASIS AND OTHER PROTOZOAL DISEASES', 'A03A-DRUGS FOR FUNCTIONAL BOWEL DISORDERS', 'S01C-ANTIINFLAMMATORY AGENTS AND ANTIINFECTIVES IN COMBINATION', 'D10A-ANTI-ACNE PREPARATIONS FOR TOPICAL USE', 'R03D-OTHER SYSTEMIC DRUGS FOR OBSTRUCTIVE AIRWAY DISEASES', 'J05A-DIRECT ACTING ANTIVIRALS', 'V03A-ALL OTHER THERAPEUTIC PRODUCTS', 'N04B-DOPAMINERGIC AGENTS', 'S03C-CORTICOSTEROIDS AND ANTIINFECTIVES IN COMBINATION', 'J04A-DRUGS FOR TREATMENT OF TUBERCULOSIS', 'S03A-ANTIINFECTIVES', 'R01A-DECONGESTANTS AND OTHER NASAL PREPARATIONS FOR TOPICAL USE', 'D06B-CHEMOTHERAPEUTICS FOR TOPICAL USE', 'L02A-HORMONES AND RELATED AGENTS', 'J04B-DRUGS FOR TREATMENT OF LEPRA', 'G03H-ANTIANDROGENS', 'A03E-ANTISPASMODICS/ANTICHOLINERGICS WITH OTHER DRUGS', 'N01B-ANESTHETICS, LOCAL', 'J01G-AMINOGLYCOSIDE ANTIBACTERIALS', 'A03C-ANTISPASMODICS IN COMBINATION WITH PSYCHOLEPTICS', 'A03B-BELLADONNA AND DERIVATIVES, PLAIN', 'L01D-CYTOTOXIC ANTIBIOTICS & RELATED SUBSTANCES', 'A01A-STOMATOLOGICAL PREPARATIONS', 'A08A-ANTIOBESITY PREPARATIONS, EXCLUDING DIET PRODUCTS', 'A12B-POTASSIUM', 'D08A-ANTISEPTICS AND DISINFECTANTS', 'D09A-MEDICATED DRESSINGS', 'J02A-ANTIMYCOTICS FOR SYSTEMIC USE', 'M01C-SPECIFIC ANTIRHEUMATIC AGENTS', 'S02A-ANTIINFECTIVES'
**Drug molecules flagged in the 30 days prior to opioid dispensation**	Gabapentin, butalbital, butorphanol, codeine, fentanyl, hydrocodone, hydromorphone, meperidine, morphine, oxycodone, pentazocine, tramadol, tapentadol, alprazolam, bromazepam, chlordiazepoxide, clobazam, clorazepate, diazepam, flurazepam, lorazepam, midazolam, nitrazepam, oxazepam, temazepam, triazolam, zolpidem, zopiclone

© 2022 Sharma V et al. *JAMA Network Open*.

**Calculated fields describing patterns of drug use**	In the previous 30 days: number of benzodiazepine/Z-drugs dispensed, number of unique benzodiazepine/Z-drug molecules dispensed, number of physicians who prescribed benzodiazepine/Z-drug and/or opioid, number of unique opioid molecules dispensed, number of opioid dispenses, number of pharmacies, oral morphine equivalents consumed, concurrent use of opioids and benzodiazepines, concurrent use of two or more opioids

*ATC code: anatomical therapeutic chemical classification codes (https://www.whocc.no/atc/structure_and_principles/)

© 2022 Sharma V et al. *JAMA Network Open*.
