# Supplementary material for: Development and Validation of a Machine Learning Model to Estimate Risk of Adverse Outcomes Within 30 Days of Opioid Dispensation
Source: JAMA Netw Open. 2022 Dec 27;5(12):e2248559. doi: 10.1001/jamanetworkopen.2022.48559 (PMC9857580; doi:10.1001/jamanetworkopen.2022.48559)
Supplement: Supplement 2. — Data Sharing Statement [file jamanetwopen-e2248559-s002.pdf]

Data Sharing Statement

Sharma. Development and Validation of a Machine Learning Model to Estimate Risk of Adverse Outcomes Within 30 Days of Opioid Dispensation. *JAMA Netw Open*. Published December 27, 2022. doi:10.1001/jamanetworkopen.2022.48559

Data
Data available: No

Additional Information
Explanation for why data not available: The data that support the findings of this study are available from Alberta Health but restrictions apply to the availability of these data, which were used under license for the current study, and so are not publicly available. However, administrative health data can be accessed from Alberta Health by following defined research protocols and confidentiality agreements.
